# Absence of Perilipin 2 Prevents Hepatic Steatosis, Glucose Intolerance and Ceramide Accumulation in Alcohol-Fed Mice

**DOI:** 10.1371/journal.pone.0097118

**Published:** 2014-05-15

**Authors:** Rotonya M. Carr, Giselle Peralta, Xiaoyan Yin, Rexford S. Ahima

**Affiliations:** 1 University of Pennsylvania, Perelman School of Medicine, Department of Medicine, Gastroenterology Division, Philadelphia, Pennsylvania, United States of America; 2 University of Pennsylvania, Perelman School of Medicine, Institute for Diabetes, Obesity and Metabolism, Philadelphia, Pennsylvania, United States of America; Boston University School of Medicine, United States of America

## Abstract

**Background:**

Perilipin 2 (Plin2) is a lipid droplet protein that has roles in both lipid and glucose homeostasis. An increase in Plin2 in liver is associated with the development of steatosis, glucose intolerance, and ceramide accumulation in alcoholic liver disease. We investigated the role of Plin2 on energy balance and glucose and lipid homeostasis in wildtype and Plin2 knockout (Plin2KO) mice chronically fed a Lieber-DeCarli liquid ethanol or control diet for six weeks.

**Methods:**

We performed *in vivo* measurements of energy intake and expenditure; body composition; and glucose tolerance. After sacrifice, liver was dissected for histology and lipid analysis.

**Results:**

We found that neither genotype nor diet had a significant effect on final weight, body composition, or energy intake between WT and Plin2KO mice fed alcohol or control diets. Additionally, alcohol feeding did not affect oxygen consumption or carbon dioxide production in Plin2KO mice. We performed glucose tolerance testing and observed that alcohol feeding failed to impair glucose tolerance in Plin2KO mice. Most notably, absence of Plin2 prevented hepatic steatosis and ceramide accumulation in alcohol-fed mice. These changes were related to downregulation of genes involved in lipogenesis and triglyceride synthesis.

**Conclusions:**

Plin2KO mice chronically fed alcohol are protected from hepatic steatosis, glucose intolerance, and hepatic ceramide accumulation, suggesting a critical pathogenic role of Plin2 in experimental alcoholic liver disease.

## Introduction

Alcoholic liver disease (ALD) is the hepatic manifestation of chronic alcohol injury, a major cause of liver failure worldwide, and the second leading indication for liver transplantation in the United States [1]. Avoidance of alcohol can ameliorate disease, but few patients are able to achieve abstinence [2], [3]. Therefore, understanding the pathogenic mechanisms of ALD is critical to addressing this public health problem. Studies have associated insulin resistance with disease severity of ALD in humans and in rodent models [4], [5], [6]; and therapies aimed at improving insulin sensitivity improve experimental ALD [7], [8]. We have demonstrated that the onset of insulin resistance is temporally related to the development of hepatic steatosis [9], an early histologic feature of ALD, thus linking hepatic steatosis with the pathogenesis of ALD.

Hepatic steatosis results from several perturbations of lipid metabolism including direct and indirect cellular injury and impairment of key lipid homeostatic pathways [10], [11], [12], [13]. The histologic focal point of hepatic steatosis is the intrahepatic lipid droplet, a dynamic organelle now recognized to have critical functions in cellular lipid homeostasis [14], [15]. Lipid droplets are comprised of cores of mostly neutral lipids (triglycerides and cholesterol esters) surrounded by a phospholipid monolayer of lipids, metabolically active enzymes, and lipid droplet proteins [16], [17], [18]. The Perilipin family of lipid droplet proteins associates with the phospholipid monolayer and we and others have shown that these proteins have roles in both lipid and glucose homeostasis in cell culture and animal models of non-alcoholic fatty liver disease [19], [20], [21], [22], [23], [24]. The role lipid droplet biology plays in the pathogenesis of ALD, however, is not well understood. In the liver, Perilipin 2 (Plin2) is the most abundant lipid droplet protein; while Perilipin 3 (Plin3) is mildly expressed and Perilipin 1 (Plin1) is *de novo* expressed in non-alcoholic steatohepatitis [25]. Plin2 is found in steroidogenic and metabolically active cells [26]; reduces the turnover of triglyceride [27]; and regulates fatty acid metabolic enzymes [24]. Furthermore, we and others have shown that Plin2 deficiency protects against diet-induced obesity and insulin resistance [20], [22], [28], [29]. The specific role of Plin2 in ALD is not known.

In ALD rodent models, Plin2 is increased in the livers of mice and rats chronically fed alcohol [19], [30], [31]. We recently reported that the increase in hepatic Plin2 is twice that of mice fed a control-liquid diet and the upregulation of Plin2 temporally coincides with the onset of hepatic steatosis, glucose intolerance and increase in hepatic ceramides (lipids that can impair insulin signaling). These findings are independent of changes in energy intake and expenditure [19] and therefore suggest an interaction between Plin2 and alcohol in lipid and glucose dysregulation. In the present study, we aimed to determine whether an absence of Plin2 prevents the development of hepatic steatosis in alcohol-fed mice. We further sought to elucidate the interactions of alcohol and Plin2 on energy, glucose and lipid homeostasis.

## Materials and Methods

### Ethics statement

Experiments were performed according to the protocols reviewed and approved by the Institutional Animal Care and Use Committee of the University of Pennsylvania. All efforts were made to minimize animal discomfort.

Adfp^Δ2–3^ mice were provided by Dr. Palczewski. Adfp^Δ2–3^ mice lack exons 2 and 3 of the *Adfp* gene but do have mRNA expression of an unstable short-form Adfp [32]. We crossed these mice from Balb/c to C57B6/J mice for ten generations and call these mice Plin2KO mice. The mice were genotyped by extracting tail DNA and using the following primers: KO forward primer CTGTCCATCTGCACGAGACTA (485 BP); WT forward primer CCCTGACTAAGACAAGGAGCA (406 bp); Common primer AGAGGGGGAACAAAAGAAAAA. Primers were mixed in a ratio of 7∶4∶3 Common∶KO∶WT. PCR reaction conditions were 94°C for 2 minutes, then 35 cycles of 94°C for 20 seconds, 57°C for 40seconds, and 72°C for 30 seconds. The final extension was 72°C for 3 minutes and PCR products were held at 15°C prior to gel analysis.

Ten-week old male C57BL/6J mice (“WT”) (Jackson Laboratory, Bar Harbor, ME) and Adfp^Δ2–3^ mice bred on C57BL/6J background (“Plin2KO”) [32] were used for the experiments. WT and Plin2KO mice were housed under ambient temperature of 22°C and 12∶12-h light-dark cycle with light on at 07:00am, and were fed either a control liquid (Ctrl) or ethanol-containing (Etoh) Lieber-DeCarli diet for six weeks. The control Lieber-DeCarli diet has 12% fat (corn oil, olive oil, and safflower oil), 70% carbohydrates (maltose-dextrin), and 18% protein calorie content. The ethanol-containing diet has ethanol added to account for 15% of total calories with the equivalent caloric amount of carbohydrates removed as previously described [9]. Mice were allowed to acclimate to the diet and were given 5% ethanol calorie content for two days then 10% ethanol calorie content for two days prior to start of the 15% ethanol calorie content diet. Groups were designated WTCtrl, WTEtoh, KOCtrl, and KOEtoh (n = 5/group). Studies were performed at one of three time points: baseline, after four weeks of feeding, or after six weeks of feeding.

### Physiologic Studies

Caloric intake was measured daily. Body weight was measured at baseline, at each increase of ethanol content, and twice a week for the duration of the study. Body composition was measured in non-fasting mice by nuclear magnetic resonance spectroscopy (Echo MRI, Houston, TX) at baseline and again at four and six weeks. Energy expenditure was measured by indirect calorimetry during a six hour fast between 10am and 4pm. Mice were allowed free access to water during fasting. Oxygen consumption (Vo_2_), carbon dioxide production (Vco_2_), respiratory quotient (RQ), and locomotor activity were measured at 4 weeks by indirect calorimetry using a Comprehensive Laboratory Animal Monitoring System (Columbus Instruments, Columbus, OH). RQ, the ratio of Vo_2_ and Vco_2_, is a measure of fuel oxidation. RQ = 0.7 indicates fat oxidation and RQ = 1 value of 1.0 indicates glucose oxidation. Locomotor activity was measured using infrared beam breaks [9], [22].

### Glucose Homeostasis

Glucose tolerance testing was performed at 4 weeks and 6 weeks in WT and Plin2KO mice. Mice were fasted for six hours (7am to 1pm) after which 2 g/kg intraperitoneal (I.P.) glucose solution was administered. Tail blood glucose was measured with a glucometer at time 0 (before glucose injection), 15, 30, 60, and 90 minutes (OneTouch Ultra; Lifescan, Inc., Milipitas, CA) [19], [21]. Mice were allowed to recover and resume their diets after completion of the testing.

### Biochemical Assays

Sixteen week old mice were euthanized at 12 noon-1pm after a four-hour fast. Blood was obtained by cardiac puncture and centrifuged to separate serum. Liver was rapidly dissected, snap frozen in liquid nitrogen, and stored at −80°C for lipid, glycogen, mRNA and protein analysis. Serum alanine aminotransferase (ALT), triglycerides, beta-hydroxybutyrate, and non-esterified fatty acids were measured using enzymatic assays [19], [21]. Liver triglyceride levels were measured after lipid extraction from livers. 100 mg of liver tissue was incubated overnight in ethanolic KOH. Tissue lysates were centrifuged and the lipid layer was separated, mixed with 1 M MgCl2, cooled on ice, and re-centrifuged. The supernatant was used for measurement of triglyceride with a standard triglyceride assay as described [9]. Ceramide analysis was done in collaboration with Dr. Michael Bennett and Jie Chen of Children's Hospital of Philadelphia. Briefly, liver samples were homogenized in Tris-EDTA lysis buffer and protein concentration was measured (Direct Detect, Millipore, Billerica, MA). C17:0 ceramide was added to 100 µL liver lysate as an internal standard. Ceramides were extracted using methyl-*tert*-butyl ether then vortexed for phase separation. The lipid layer was isolated, dried and reconstituted in a methanol/chloroform solution for flow injection tandem mass spectrometer analysis [33], [34].

RNA was extracted from livers using TRIzol reagent (Invitrogen, Carlsbad, CA), and expression of mRNA levels of Plin1, Plin2, and Plin3 and lipogenic and lipolytic enzymes were measured with real-time PCR (ABI Prism; Applied Biosystems, Foster City, CA). The level of mRNA expression was normalized to phosphoriboprotein (36B4) [9].

Insulin was measured using a standard ELISA kit according to manufacturer's instructions (Crystal Chem (Evanston, IL) [19].

### Immunoblotting

Hepatic Plin2 protein levels were determined by immunoblotting. 20 mg of liver tissue were homogenized in lysis buffer containing 1% NP-40, 0.5% Triton, 10% glycerol, 0.15 M NaCl, 0.001 M EDTA, 0.5 M Tris·HCl, at pH 7.4, and supplemented with complete protein inhibition cocktail tablet from Roche (Penzberg, Germany). 30 mcg of protein were separated by 4–12% NuPage Bis-Tris gel (Invitrogen, Grand Island, NY) and wet transferred to nitrocellulose membranes overnight at 4°C. Membranes were blocked with 5% milk for 1 hour at room temperature and incubated with guinea pig polyclonal antibody against Plin2 (Fitzgerald, Concord, MA) at 1∶1000 dilution overnight at 4C. After serial washes, horseradish peroxidase-conjugated goat anti-guinea pig secondary antibody was applied at 1∶5000 dillution at room temperature for one hour followed by washing and visualization with enhanced chemiluminescence (GE Healthcare, Piscataway, NJ). Membranes were stripped and blotted for GAPDH at 1∶1000 dilution (Cell Signaling, Beverly, MA) [19].

### Histology

Liver samples were excised from the right hepatic lobe, placed in a cartridge and fixed in 10% buffered formalin overnight and submitted to pathology core for hematoxylin and eosin staining. Slides were visualized under bright field with Nikon 80i microscope. Images were captured with a Nikon DS-Qi1MC camera and image analysis system (Nikon Instruments, Melville, NY).

### Statistics

Data are expressed as means +/− SEM. T-test or analysis of variance (ANOVA) and post-hoc Newman-Keuls multiple comparison test (GraphPad Prism, La Jolla, CA) were used for statistical analysis. p<0.05 is considered significant.

## Results

### Effects of Plin2 deficiency and alcohol on energy balance

**Figure 1:**
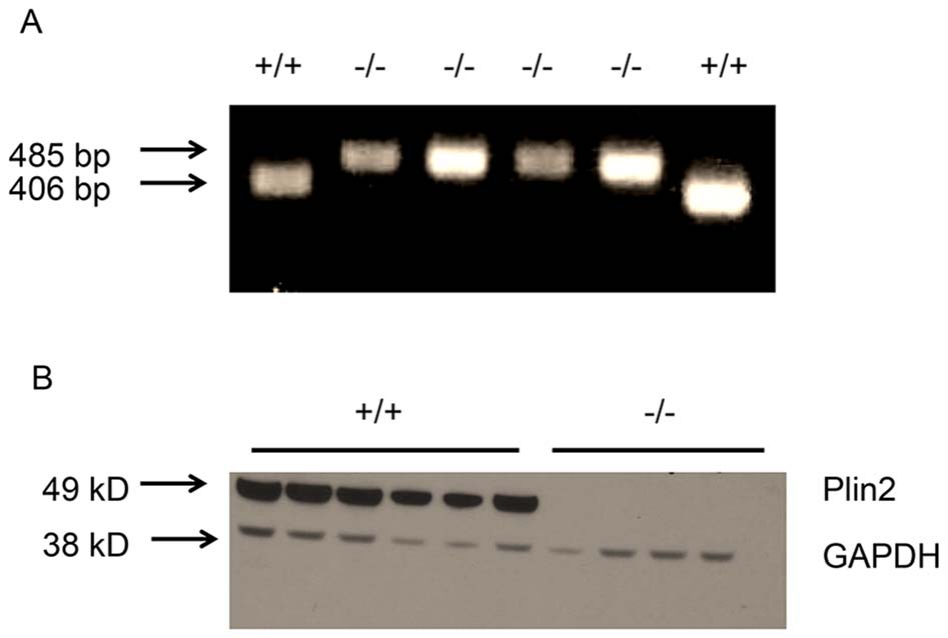
Confirmation of plin2ko mice. A) Genotyping of wildtype +/+ and Plin2KO (−/−) mice using tail DNA. Bands correspond to 485 BP KO PCR product and 406 bp WT PCR product. B) Immunoblots of Plin2 and GAPDH from liver lysates of wildtype and Plin2KO mice. Each lane represents liver lysate from an individual mouse. Plin2 = Perilipin 2 GAPDH =  glycerol-3-phosphate dehydrogenase housekeeping protein.

Wildtype (WT) and Plin2KO (KO) mice were confirmed by genotyping and immunoblot of liver lysates (**Fig. 1A–B**). Despite similar baseline total body weight, the WT mice had higher baseline total fat mass compared with KO mice (2.5 g vs 1.6 g, p = 0.008). At four weeks and six weeks, there was an increase in fat mass in all groups. Neither Plin2 deficiency nor alcohol had a significant effect on the final weight or body composition (**Table 1**).

**Table 1:** Metabolic Phenotyping of Wildtype and Plin2KO Mice Fed Control or Ethanol Diets.

	WTCtrl	WTEtoh	KOCtrl	KOEtoh	p
Metabolic parameters					
Baseline weight (g)	22.6±1.08	24.22±1.12	22.66±0.72	23.48±0.35	0.53
Final weight (g)	28.7±1.30	28.94±1.18	28.76±0.73	27.2±0.46	0.58
Energy intake (g/mouse/d)	11.53±0.83	12.06±1.26	11.89±0.86	11.47±1.25	0.16
Baseline fat (g)	2.26±0.13	2.66±0.28	1.66±0.10*^ 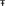 ^	1.48±0.14*^ 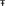 ^	**0.008**
Baseline lean (g)	20.01±0.85	20.86±0.83	19.67±0.73	20.68±0.49	0.64
4 week fat (g)	4.66±0.40	4.04±0.55	4.14±0.55	2.79±0.43	0.08
4 week lean (g)	20.71±1.02	22.58±0.58	20.51±0.65	20.80±0.70	0.23
Final fat (g)	6.11±0.55	5.86±0.81	5.97±0.74	4.49±0.68	0.36
Final lean (g)	22.56±1.06	23.77±0.62	22.56±0.65	22.05±0.55	0.43
Vo_2_ (ml/h)	87.72±5.31	86.04±3.87	119.8±9.34**^§^	113.4±10.78***	**0.04**
Vco_2_ (mL/h)	61.9±4.17	60.36±2.81	84.26±6.94**^§^	80.64±8.40	**0.04**
RQ	0.70±0.005	0.70±0.004	0.70±0.004	0.70±0.007	0.96
Activity (counts)	9754±1727	14531±1619	10725±1510	11862±1114	0.18

Vo_2_ = oxygen consumption; Vco_2_ = carbon dioxide production Ctrl = control diet Etoh = ethanol diet; WT = wildtype; KO = Plin2 KO. Data reported are mean ±SEM and compared by ANOVA. p<0.05 is considered significant. *p<0.05 vs WTCtrl; **p = 0.03 vs WTCtrl; ***p = 0.04 vs WTCtrl; ^
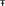
^p<0.01 vs WTEtoh; ^§^p = 0.04 vs WTEtoh.

Energy homeostasis is a balance between energy intake and expenditure. The caloric intake was similar in all groups (**Table 1**). Energy expenditure was assessed by indirect calorimetry. There was no difference in locomotor activity and fat oxidation. However, KO mice on control diet had higher oxygen consumption (Vo_2_) (117 vs 87 ml/h, p = 0.04) and carbon dioxide production (Vco_2_) (82 vs 61 ml/h, p = 0.04) than WT mice. Alcohol feeding did not affect Vo_2_ or Vco_2_ in WT or Plin2KO mice (**Table 1**).

### Effects of Plin2 deficiency on glucose homeostasis in alcohol-fed mice

**Figure 2:**
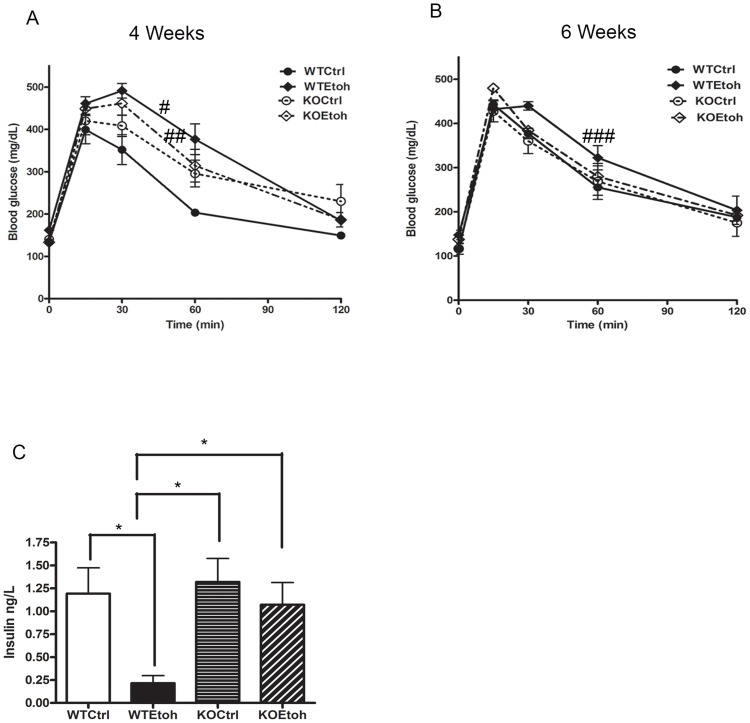
Measurement of glucose tolerance in wildtype and Plin2KO (KO) mice fed control liquid or ethanol diet. Glucose tolerance test after A) 4 weeks and B) 6 weeks of feeding. # P = 0.001 WTCtrl vs WTEtoh; ##P = 0.03 WTCtrl vs KOEtoh; ###P<0.05 WTCtrl vs WTEtoh; C) Serum insulin levels at 6 weeks. *P<0.05. N = 5 mice/group. P<0.05 is considered significant.

We previously reported glucose intolerance develops in alcohol-fed WT mice at four weeks of feeding and coincides with an upregulation of hepatic Plin2 [9]. We therefore examined the effects of alcohol on glucose tolerance in Plin2KO mice. WT and Plin2KO mice fed either a control liquid diet or alcohol diet underwent a glucose tolerance test (GTT) at four weeks and at six weeks. Baseline weight and glucose levels were similar in both groups prior to the GTT. WT mice fed alcohol had impaired glucose tolerance at both 4 weeks and 6 weeks compared with WT mice fed control diets. There was no difference, however, in glucose tolerance between Plin2KO mice fed control or alcohol diets (**Fig. 2A–B**). In addition, serum insulin levels were significantly reduced in WTEtoh mice compared with WTCtrl mice suggesting impairment of pancreatic islet function. Mice lacking Plin2, however, had similar insulin levels as WTCtrl mice regardless of alcohol feeding (**Fig. 2C**). Thus, alcohol feeding failed to impair glucose tolerance in the absence of Plin2.

### Effects of Plin2 deficiency on serum and hepatic lipids in alcohol-fed mice

**Figure 3:**
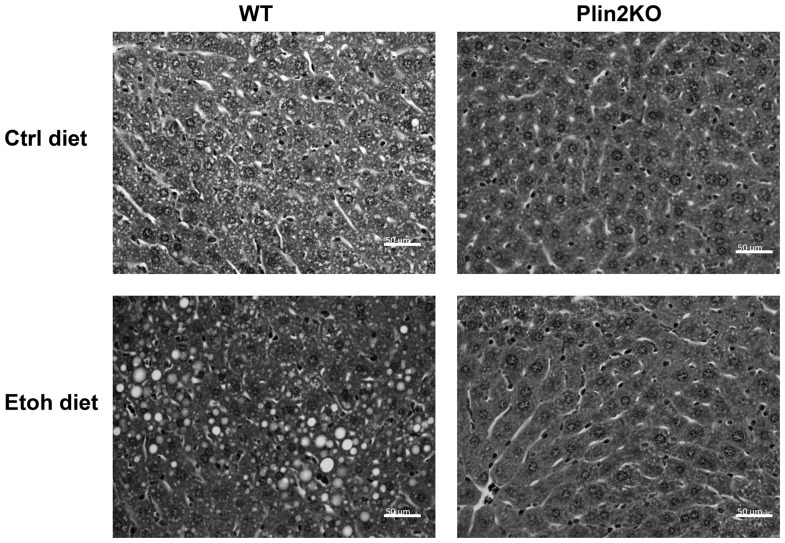
Liver histology of wild-type and Plin2KO mice fed control liquid or ethanol diet for 6 weeks. Representative hematoxylin and eosin-stained liver sections. 40x magnification. WT = wildtype KO = Plin2KO Ctrl = control Etoh = ethanol

We next examined liver histology and measured serum and hepatic lipids to determine effects of alcohol on lipid homeostasis in the absence of Plin2. In agreement with previous studies, WTEtoh mice developed hepatic steatosis compared with WTCtrl mice. KOCtrl mice had no histologic evidence of hepatic steatosis and had reduced hepatic triglycerides compared with WT mice. Liver histology in KOEtoh mice was consistent with the lack of steatosis (**Fig. 3**).

**Figure 4:**
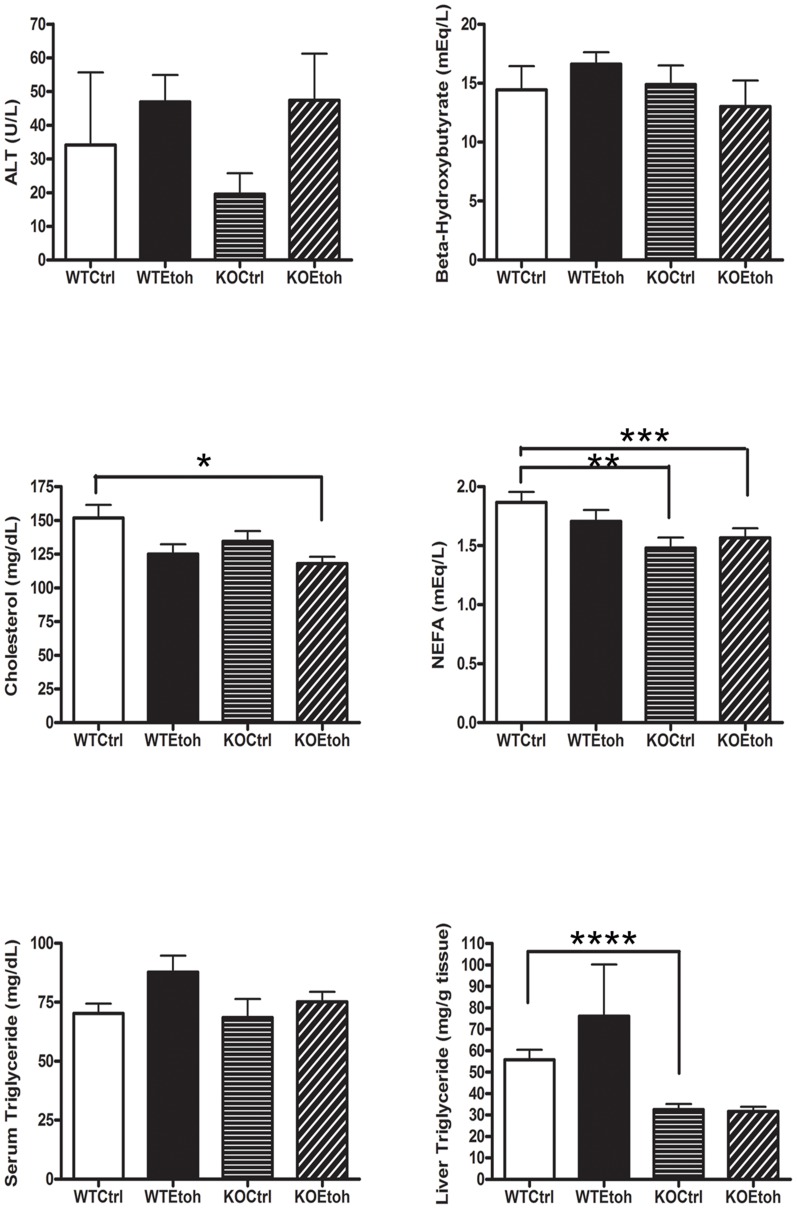
Serum and liver chemistries of wildtype and Plin2KO mice fed control or ethanol diets. Graphs depict results of serum and liver assays on wildtype (WT) and Plin2KO (KO) mice after six weeks of feeding a control (Ctrl) or ethanol (Etoh) diet. N = 5 mice/group. Data reported are mean ±SEM. *p = 0.01 **p = 0.02 ***p = 0.04 ****p = 0.002. p<0.05 is considered significant.

Analysis of serum lipids revealed that serum triglyceride (TG) was mildly, but non-statistically elevated in WTEtoh and KOEtoh mice compared with their control-fed counterparts; and serum cholesterol levels were lower in WTEtoh and KOEtoh groups than in control-fed mice (p = 0.03). Non-esterified fatty acid levels were significantly higher in WT mice than in KO mice (1.8 vs 1.5 mEq/L, p = 0.04) but independent of effects of diet (**Fig. 4**). These results suggest that neither reduced circulating triglycerides nor fatty acids account for the lack of effect of alcohol on hepatic triglyceride accumulation in the absence of Plin2.

**Figure 5:**
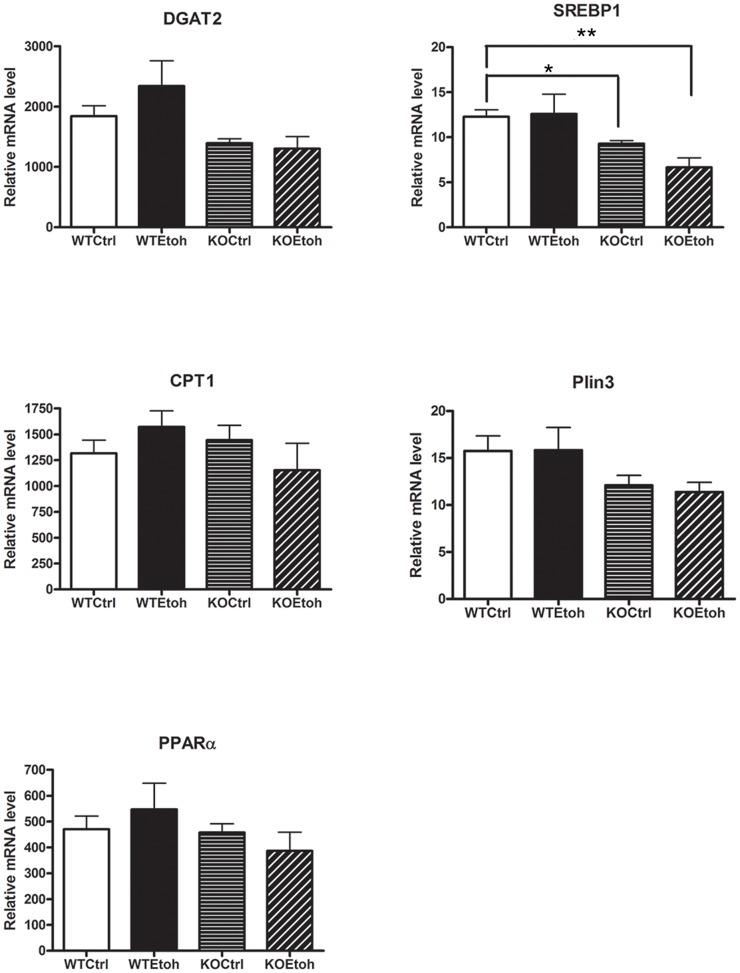
Liver mRNA of wildtype and Plin2KO mice fed control or ethanol diets. Hepatic mRNA expression relative to 36B4 (phosphoriboprotein) using liver lysates of wildtype (WT) and Plin2KO (KO) mice fed six weeks of control (Ctrl) or ethanol (Etoh) diets. N = 5 mice/group. DGAT2 =  diacylglycerol O-acyltransferase 2; CPT1 =  Carnitine palmitoyltransferase I; PPARα =  peroxisome proliferator-activated receptor alpha; SREBP1 = Sterol regulatory element-binding protein 1; Plin3 =  Perilipin 3. Data reported are means ±SEM. *p = 0.02 **p = 0.003. p<0.05 is considered significant.

To further examine the mechanisms of reduced hepatic triglyceride content in Plin2KO mice, we examined expression of a number of key regulators of lipid synthesis, metabolism and storage. mRNA analysis suggests significant downregulation of SREBP1 (a lipogenic transcription factor) in KOEtoh mice compared with WTCtrl and WTEtoh mice; and a downward trend of DGAT2 (a key enzyme in the triglyceride synthetic pathway) in KOCtrl and KOEtoh mice compared with WT groups. Plin1 and Plin3 expression was not altered in Plin2KO indicating a lack of developmental compensation for the absence of Plin2 (**Fig. 5**).

### Effects of Plin2 deficiency on hepatic ceramide content in alcohol-fed mice

**Figure 6:**
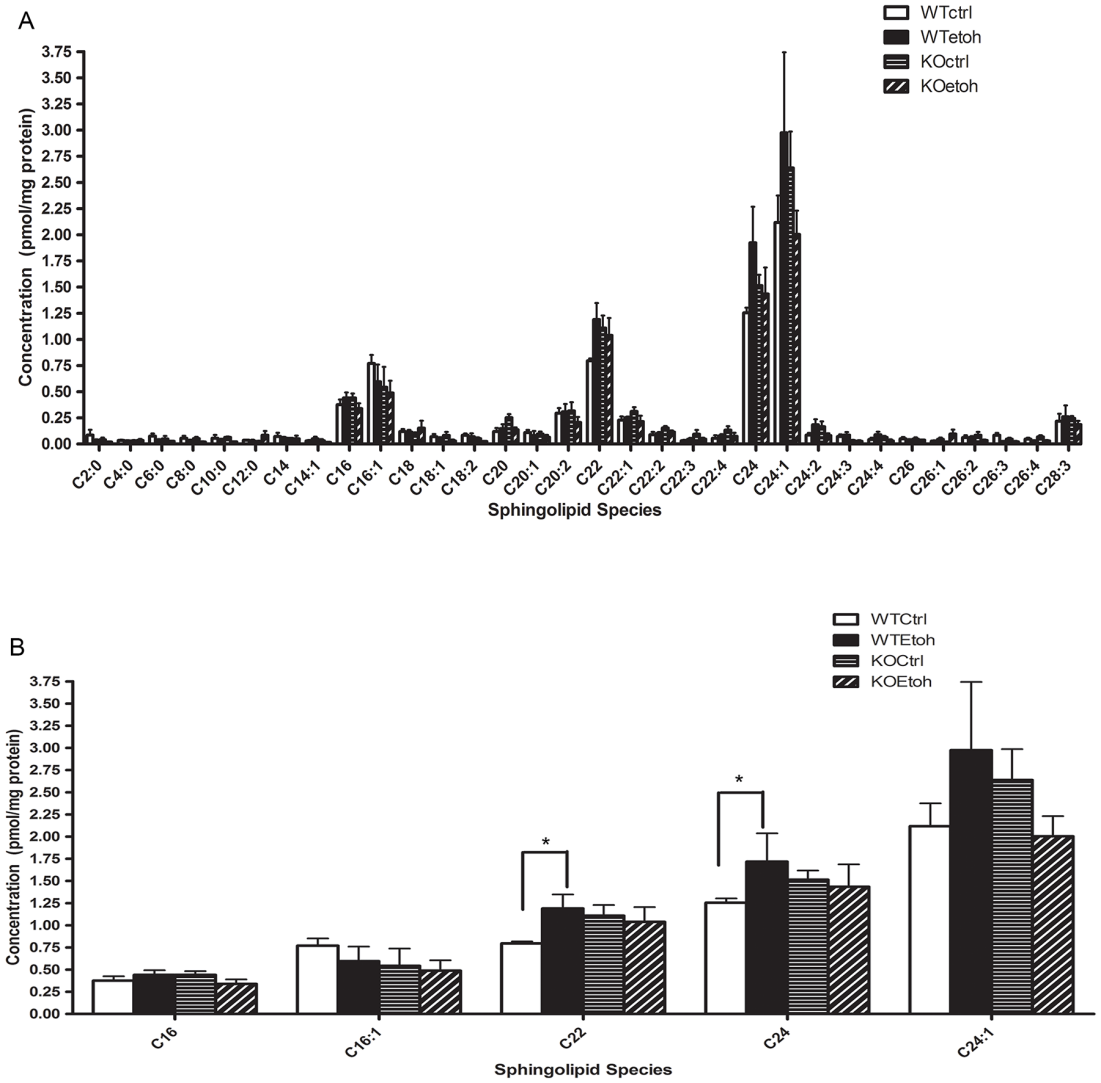
Hepatic ceramide measurements in WT and Plin2KO mice fed control or ethanol diets. A) Distribution of short (C2-C5), medium (C6-C12), long (C13-C21) and very long chain (>21) hepatic ceramides as measured by mass spectrometric analysis in WT and Plin2KO (KO) mice fed control (Ctrl) or ethanol (Etoh) diets for six weeks B) Comparison of predominant ceramide species in WT and KO mice fed control or ethanol diets. N = 5 mice/group. Data reported are mean ±SEM. *p<0.05.

Ceramides are lipid metabolites that are upregulated in both human and rodent ALD [35], [36], [37], [38], can impair insulin signaling [39], and can derive from sphingomyelin, a sphingolipid component of the lipid droplet membrane [17]. We previously demonstrated a temporal relationship between the development of hepatic steatosis, upregulation of Plin2 and accumulation of hepatic ceramides in alcohol-fed mice [9]. Therefore, we examined the effect of alcohol on hepatic ceramides in the absence of Plin2. We measured short (C2-C5), medium (C6-C12), long (C13-21) and very long-chain (>21) ceramide species in WT and Plin2KO mice fed control and alcohol diets and observed that similar to our published results of WT mice [9], long and very long-chain ceramides were the most abundant in both alcohol and control-fed mice. In particular, we noted that C16, C16.1, C22, C24 and C24:1 were the predominant ceramide species (**Fig 6A**). Alcohol feeding significantly increased C22 and C24; however, in the absence of Plin2 alcohol failed to increase hepatic ceramides (**Fig. 6B**), suggesting Plin2 may regulate ceramide synthesis in alcohol-fed mice.

## Discussion

A key feature of ALD is impaired hepatic lipid metabolism. Dysregulated lipid metabolism in ALD results from multiple mechanisms including: upregulation of inflammatory pathways which result in increased cytokine production [40], [41], cellular oxidative stress and impaired cellular lipid homeostasis [42]; inhibition of fatty acid beta oxidation through downregulation of beta-oxidative enzymes [10] and direct mitochondrial injury [43]; and direct and indirect upregulation of key cellular lipogenic pathways [11], [44].

The net effect of alcohol's effects on hepatic lipid metabolism is hepatic steatosis, an accumulation of intra-hepatocellular lipid droplets, which are metabolically active organelles associated with Perilipin family lipid droplet proteins [16], [17]. There are five known members of the Perilipin family of proteins differing in distribution and function, and in the liver, Plin2 predominates [25]. Plin2 is upregulated in experimental models of ALD [9], [30], [31], is a reliable marker of steatosis in alcohol fed rats [30], and coats large lipid droplets in mice fed a high fat, ethanol diet [31]. Plin2 is also upregulated in WIF-B cells treated with oleate and ethanol [45]. Plin2 deficiency improves hepatic steatosis and insulin sensitivity in a diet-induced obesity model of hepatic steatosis [22], [28] and is increased in hepatic LDs in response to high fat refeeding [18], but to our knowledge, this is the first study that has examined the role of Plin2 in the pathogenesis of ALD *in vivo*.

Here, we demonstrate that absence of Plin2 prevents alcohol-mediated dysregulation of hepatocellular lipid homeostasis. In contrast to their wild-type littermates, Plin2KO mice fed alcohol do not develop hepatic steatosis despite the presence of Plin3 (a lipid droplet protein shown to compensate for Plin2) [46]. Moreover, hepatic triglyceride levels are dramatically reduced in Plin2KOEtoh mice compared with WTEtoh mice. Although the difference in liver triglyceride levels did not reach statistical significance, this lack of significance is likely due to the high degree of variability in steatosis in WTEtoh mice. The lack of hepatic steatosis and reduced hepatic triglyceride levels in Plin2KO mice result from multifactorial effects on cellular lipid homeostasis, namely, a downregulation of *de novo* lipogenic and triglyceride synthetic pathways and a lack of compensation for Plin2 by Plin1 and Plin3. Indeed, Plin2 has been shown to prevent lipolysis of lipid droplets and reduce triglyceride turnover [27]; and a human Plin2 polymorphism has been linked with lipid droplet stabilization and reduced lipolysis [47].

Several experimental models demonstrate that other Perilipin lipid droplet protein family members can compensate for Plin2 deficiency; therefore the lack of compensation by Plin3 for Plin2 was unexpected. Plin3 compensates for Plin2 deficiency in macrophages, murine adipocytes, and Adfp-null adipocytes [46], [48], [49]. We did not, however, observe compensation for Plin3 by Plin2 in the livers of mice fed a high fat diet [19], suggesting that differences in findings may depend on the models and tissues studied. Lack of compensation of Plin1 for Plin2, however, was expected. In our recent study, Plin1 is barely detectable [9] and to our knowledge, *de novo* expression of hepatic Plin1 has only been reported in non-alcoholic steatohepatitis [25]. Future evaluation of other Perilipin protein members may yield additional insights into the relationship of other lipid droplet proteins with ALD pathogenesis.

The effects on lipid homeostasis were independent of changes in energy intake and expenditure. Both WT and KO mice have equivalent food intake and activity level and utilize fatty acids as the primary fuel while fasting. We did note increased oxygen consumption and carbon dioxide production in Plin2KO mice compared with WT mice independent of effects of alcohol. These findings likely result from compensation from a reduced ability to store fuel as fat; indeed, baseline fat stores are lower in Plin2KO mice than in WT mice.

Impaired glucose intolerance is the clinical result of insulin resistance, develops at the onset of hepatic steatosis in mice chronically fed alcohol [9], and has been linked with disease pathogenesis in human and experimental ALD [4], [5], [6], [9]. An advantage of using the mouse Lieber-DeCarli liquid alcohol feeding model to examine the effect of hepatic steatosis on glucose homeostasis is that mice fed this diet do not develop significant steatohepatitis or fibrosis [50], [51], disease states that are independently associated with insulin resistance [4]. We can therefore examine the role of lipid droplet biology *per se* in the pathogenesis of insulin resistance in ALD. While we did not directly test insulin sensitivity here, our findings suggest improvement of hepatic insulin sensitivity, since absence of Plin2 prevents alcohol-induced glucose intolerance and restores glycogen stores (data not shown) to levels of control-fed WT mice. Importantly, Plin2KO mice fed alcohol do not have impaired insulin secretion as in WT mice fed alcohol. Impaired insulin secretion has been previously observed in isolated perfused pancreas of alcohol fed rats [52], [53]. Thus, impaired insulin secretion in response to alcohol and reduced glucose disposal may both contribute to the impaired glucose tolerance demonstrated in this study. Our data suggest that Plin2 deficiency prevents these alcohol mediated effects on glucose tolerance. Our future studies will investigate specific mechanisms by which Plin2 deficiency protects against pancreatic beta cell dysfunction and glucose intolerance.

Bioactive lipid metabolites can impair insulin signaling. In ALD, ceramides (sphingolipid metabolites) accumulate in the livers of both humans and rodents with ALD and are implicated in disease severity [35], [37], [54]. Ceramide biosynthesis and metabolism is complex and involves three major synthetic pathways and metabolism to other sphingolipid species. Through mechanisms that are incompletely understood, ceramide accumulation results in activation of protein phosphatase 2A (PP2A) and subsequent inhibition of AKT phosphorylation, thereby impairing insulin signaling [39], [55], [56]. The ceramide precursor sphingomyelin is a component of the lipid droplet membrane and the production of ceramide from sphingomyelin hydrolysis is implicated in alcohol's impairment of glucose homeostasis [54]. Here, we show a predominance of C16, C16.1, C22, C24 and C24:1 ceramides with alcohol-feeding. Little is known about the role of specific ceramide species in ALD, but reduction of C24 in alcohol fed mice has been shown to improve hepatic steatosis [54]. Studies in other disease states have shown that C22 may have anti-proliferative properties [56]; C24 and C24:1 have pro-proliferative properties [57]; and C16 may promote apoptosis [58], [59], thus its accumulation may conceivably promote alcohol-induced hepatotoxicity.

Pharmacologic inhibition of ceramide de novo synthesis with myriocin improves hepatic insulin signaling in Long-Evans rats chronically fed alcohol [60] and we previously reported that the onset of hepatic steatosis and insulin resistance in experimental ALD temporally correlates with an increase in long-chain hepatic ceramides and upregulation of Plin2 [9]. Our current results show that the increases in hepatic ceramides are prevented in the absence of Plin2 suggesting that Plin2 may mediate both cellular ceramide metabolism and insulin resistance in ALD, thus making Plin2 a potential target for therapy and/or prevention of ALD.

In summary, alcohol-fed Plin2KO mice are protected from hepatic steatosis, glucose intolerance and hepatic ceramide accumulation. These results suggest a distinct role of Plin2 in the pathogenesis of ALD and ceramide metabolism and highlight the importance of additional studies to understand the specific mechanisms that link Plin2 to the pathogenesis of ALD. Future studies will additionally investigate the role of Plin2 in advanced stages of alcoholic liver disease.
